# Poly[diaqua-μ_2_-oxalato-di-μ_4_-terephthalato-dilutetium(III)]

**DOI:** 10.1107/S1600536809034370

**Published:** 2009-09-05

**Authors:** Zhi-Feng Li, Chun-Xiang Wang

**Affiliations:** a School of Materials & Chemical Engineering, Jiangxi University of Science and Technology, Ganzhou 341000, People’s Republic of China

## Abstract

In the title compound, [Lu_2_(C_8_H_4_O_4_)_2_(C_2_O_4_)(H_2_O)_2_]_*n*_, the Lu^3+^ cations are each coordinated by eight O atoms of four terephthalate anions, one oxalate anion and one aqua ligand to complete a distorted square-anti­prismatic geometry. They are bridged by the terephthalate ligands, generating a three-dimensional framework, which is further stabilized by the oxalate ligands. The terephthalate ions and oxalate ions are all located on centers of inversion.

## Related literature

For bond lengths and angles in terephthalate anions, see: Daiguebonne *et al.* (2006 ▶).
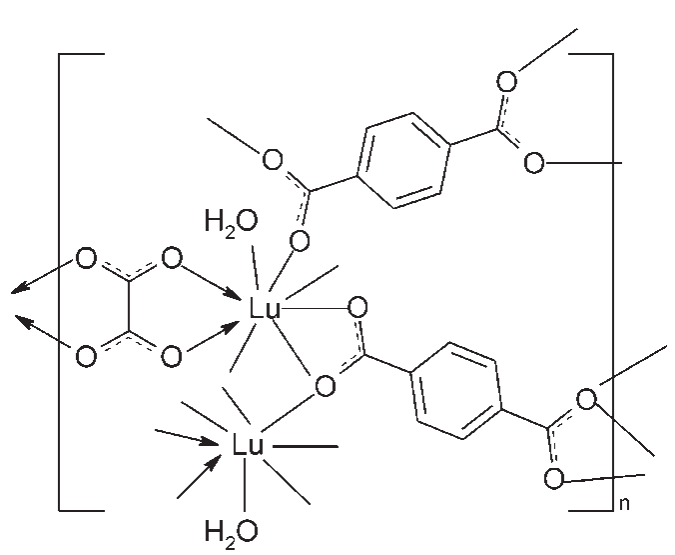


## Experimental

### 

#### Crystal data

[Lu_2_(C_8_H_4_O_4_)_2_(C_2_O_4_)(H_2_O)_2_]
*M* *_r_* = 802.22
Triclinic, 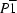

*a* = 7.0020 (4) Å
*b* = 7.5750 (4) Å
*c* = 10.2068 (6) Å
α = 75.472 (1)°
β = 70.843 (1)°
γ = 88.255 (1)°
*V* = 494.24 (5) Å^3^
*Z* = 1
Mo *K*α radiation
μ = 10.01 mm^−1^
*T* = 295 K
0.12 × 0.09 × 0.06 mm

#### Data collection

Bruker APEXII CCD area-detector diffractometer
Absorption correction: multi-scan (*SADABS*; Sheldrick, 1996 ▶) *T*_min_ = 0.348, *T*_max_ = 0.542
2812 measured reflections
1962 independent reflections
1850 reflections with *I* > 2σ(*I*)
*R*_int_ = 0.011

#### Refinement

*R*[*F* ^2^ > 2σ(*F* ^2^)] = 0.016
*wR*(*F* ^2^) = 0.038
*S* = 1.09
1962 reflections
154 parameters
H-atom parameters constrained
Δρ_max_ = 0.93 e Å^−3^
Δρ_min_ = −1.28 e Å^−3^

### 

Data collection: *APEX2* (Bruker, 2007 ▶); cell refinement: *SAINT* (Bruker, 2007 ▶); data reduction: *SAINT*; program(s) used to solve structure: *SHELXS97* (Sheldrick, 2008 ▶); program(s) used to refine structure: *SHELXL97* (Sheldrick, 2008 ▶); molecular graphics: *SHELXTL* (Sheldrick, 2008 ▶); software used to prepare material for publication: *SHELXTL*.

## Supplementary Material

Crystal structure: contains datablocks I, global. DOI: 10.1107/S1600536809034370/nk2003sup1.cif

Structure factors: contains datablocks I. DOI: 10.1107/S1600536809034370/nk2003Isup2.hkl

Additional supplementary materials:  crystallographic information; 3D view; checkCIF report

## Figures and Tables

**Table 1:** Selected bond lengths (Å)

Lu—O1	2.825 (3)
Lu—O1^i^	2.304 (3)
Lu—O2	2.297 (2)
Lu—O3	2.259 (2)
Lu—O4^ii^	2.195 (2)
Lu—O5	2.303 (3)
Lu—O6^iii^	2.313 (3)
Lu—O7	2.272 (3)

Symmetry codes: (i) 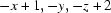; (ii) 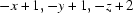; (iii) 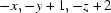.

**Table 2:** Hydrogen-bond geometry (Å, °)

*D*—H⋯*A*	*D*—H	H⋯*A*	*D*⋯*A*	*D*—H⋯*A*
O7—H7*A*⋯O3^i^	0.85	1.92	2.752 (5)	167
O7—H7*B*⋯O2^iv^	0.85	1.92	2.764 (6)	177

Symmetry codes: (i) 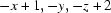; (iv) 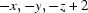.

**Fig. 1.**
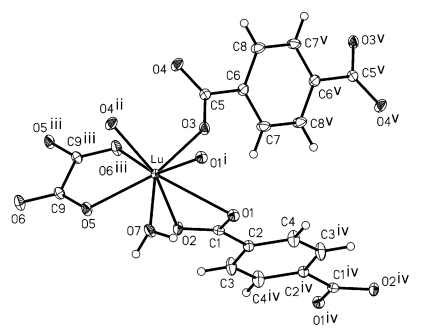
The asymmetric unit of the title compounds, with the atom-numbering scheme. Displacement ellipsoids are drawn at the 30% probability level and H atoms are shown as small spheres of arbitrary radii. Symmetry code: (i) 1 - x, -y, 2 - z; (ii) 1 - x, 1 - y, 2 - z; (iii) -x, 1 - y, 2 - z; (iv) 1 - x, -y, 1 - z; (v) 2 - x, 1 - y, 1 - z.

## supplementary crystallographic information

### Comment

In the title compound, the asymmetric unit consists of one Lu^3+^ cation, one half of oxalate anion, two half of terephthalate anions and one aqua ligand. The Lu atoms are each coordinated by eight O atoms of four terephthalate anions, one oxalate anion and one aqua ligand to complete a distorted square antiprismatic geometry (Fig.1). The Lu–O distances are in the range of 2.195 (2)–2.825 (3) Å. The two crystallography independent terephthalate (tp) anions are both located on the center of symmetry and exhibit different types of coordination mode to Lu atoms. The tp1 (O1 to O2, C1 to C4) anion functions as chelating-bridging tridentate ligand, two carboxylate oxygen atoms chelate one Lu atom in which one oxygen atom additionally bonded to another Lu atom with the Lu···Lu seperation of 4.245 (2) Å. Then two edge-shared [LuO_8_] polyhedra are bridged by the bidentate tp2 (O3 to O4, C5 to C8) ligands to generate one-dimensional chains along [010] direction. Thus the chains are linked by the tp1 and tp2 ligands into a three-dimensional framework. Bond lengths and angles within the terephthalate anions exhibit normal values (Daiguebonne *et al.*, 2006). The oxalate ions are also located on centers of inversion and act as double bidentate (tetradentate) ligand in the linear chain which connect the edge-shared [LuO_8_] polyhedra along [100] direction to stabilize the three-dimensional framework. The aqua ligands donate hydrogen atoms to terephthalate oxygen atoms O2 and O3 to form hydrogen bonds.

### Experimental

A mixture of LuCl_3_.6H_2_O (1.00 mmol, 0.39 g), oxalic acid (0.50 mmol, 0.05 g), terephthalic acid (0.50 mmol, 0.09 g), NaOH (2.00 mmol, 0.08 g) and H_2_O (10.0 ml) was heated in a 23 ml stainless steel reactor with a Teflon liner at 443 K for 48 h. A small amount of colorless column-like crystals were filtered and washed with water and acetone.

### Refinement

H atoms attached to C atoms were included at calculated positions and treated as riding atoms [C—H = 0.93 Å and *U*_iso_(H) = 1.2*U*_eq_(C)]. The water H atoms were found in a difference map, relocated in idealized positions (O—H = 0.85 Å) and refined as riding atoms with *U*_iso_(H) = 1.5*U*_eq_(O). The highest density peak and deepest hole are located 0.88 Å and 0.90 Å from atom Lu.

### Crystal data

**Table:**

[Lu_2_(C_8_H_4_O_4_)_2_(C_2_O_4_)(H_2_O)_2_]	*Z* = 1
*M**_r_* = 802.22	*F*(000) = 374
Triclinic, *P*1	*D*_x_ = 2.695 Mg m^−^^3^
Hall symbol: -P 1	Mo *K*α radiation, λ = 0.71073 Å
*a* = 7.0020 (4) Å	Cell parameters from 368 reflections
*b* = 7.5750 (4) Å	θ = 1.7–26.8°
*c* = 10.2068 (6) Å	µ = 10.01 mm^−^^1^
α = 75.472 (1)°	*T* = 295 K
β = 70.843 (1)°	Rod, colourless
γ = 88.255 (1)°	0.12 × 0.09 × 0.06 mm
*V* = 494.24 (5) Å^3^	

### Data collection

**Table:**

Bruker APEXII CCD area-detector diffractometer	1962 independent reflections
Radiation source: fine-focus sealed tube	1850 reflections with *I* > 2σ(*I*)
graphite	*R*_int_ = 0.011
φ and ω scans	θ_max_ = 26.5°, θ_min_ = 3.1°
Absorption correction: multi-scan (*SADABS*; Sheldrick, 1996)	*h* = −8→8
*T*_min_ = 0.348, *T*_max_ = 0.542	*k* = −9→9
2812 measured reflections	*l* = −8→12

### Refinement

**Table:**

Refinement on *F*^2^	Primary atom site location: structure-invariant direct methods
Least-squares matrix: full	Secondary atom site location: difference Fourier map
*R*[*F*^2^ > 2σ(*F*^2^)] = 0.016	Hydrogen site location: inferred from neighbouring sites
*wR*(*F*^2^) = 0.038	H-atom parameters constrained
*S* = 1.09	*w* = 1/[σ^2^(*F*_o_^2^) + (0.0189*P*)^2^ + 0.6568*P*] where *P* = (*F*_o_^2^ + 2*F*_c_^2^)/3
1962 reflections	(Δ/σ)_max_ = 0.001
154 parameters	Δρ_max_ = 0.93 e Å^−^^3^
0 restraints	Δρ_min_ = −1.28 e Å^−^^3^

### Special details

**Table:**

Geometry. All e.s.d.'s (except the e.s.d. in the dihedral angle between two l.s. planes) are estimated using the full covariance matrix. The cell e.s.d.'s are taken into account individually in the estimation of e.s.d.'s in distances, angles and torsion angles; correlations between e.s.d.'s in cell parameters are only used when they are defined by crystal symmetry. An approximate (isotropic) treatment of cell e.s.d.'s is used for estimating e.s.d.'s involving l.s. planes.
Refinement. Refinement of *F*^2^ against ALL reflections. The weighted *R*-factor *wR* and goodness of fit *S* are based on *F*^2^, conventional *R*-factors *R* are based on *F*, with *F* set to zero for negative *F*^2^. The threshold expression of *F*^2^ > σ(*F*^2^) is used only for calculating *R*-factors(gt) *etc*. and is not relevant to the choice of reflections for refinement. *R*-factors based on *F*^2^ are statistically about twice as large as those based on *F*, and *R*- factors based on ALL data will be even larger.

### Fractional atomic coordinates and isotropic or equivalent isotropic displacement parameters (Å^2^)

**Table:**

	*x*	*y*	*z*	*U*_iso_*/*U*_eq_	
Lu	0.30978 (2)	0.22275 (2)	1.015515 (15)	0.01188 (6)	
C1	0.3866 (5)	0.0271 (5)	0.7936 (4)	0.0153 (7)	
C2	0.4422 (6)	0.0079 (5)	0.6429 (4)	0.0152 (7)	
C3	0.3265 (6)	0.0825 (6)	0.5581 (4)	0.0291 (10)	
H3	0.2092	0.1391	0.5967	0.035*	
C4	0.6172 (6)	−0.0741 (6)	0.5833 (4)	0.0281 (9)	
H4	0.6975	−0.1239	0.6385	0.034*	
C5	0.7147 (5)	0.4796 (5)	0.7823 (4)	0.0158 (7)	
C6	0.8631 (6)	0.4908 (5)	0.6353 (4)	0.0173 (7)	
C7	0.8285 (6)	0.3850 (6)	0.5519 (4)	0.0254 (9)	
H7	0.7123	0.3078	0.5871	0.030*	
C8	1.0350 (6)	0.6072 (6)	0.5834 (4)	0.0242 (9)	
H8	1.0586	0.6793	0.6387	0.029*	
C9	−0.0823 (5)	0.4315 (5)	1.0581 (4)	0.0169 (7)	
O1	0.4816 (4)	−0.0502 (4)	0.8752 (3)	0.0195 (6)	
O2	0.2488 (4)	0.1349 (4)	0.8326 (3)	0.0194 (6)	
O3	0.6143 (4)	0.3288 (3)	0.8501 (3)	0.0178 (5)	
O4	0.7003 (4)	0.6170 (4)	0.8308 (3)	0.0217 (6)	
O5	−0.0308 (4)	0.2714 (4)	1.0930 (3)	0.0219 (6)	
O6	−0.2519 (4)	0.4916 (4)	1.1081 (3)	0.0219 (6)	
O7	0.1348 (4)	−0.0421 (4)	1.1585 (3)	0.0217 (6)	
H7B	0.0166	−0.0741	1.1644	0.032*	
H7A	0.1966	−0.1407	1.1670	0.032*	

### Atomic displacement parameters (Å^2^)

**Table:**

	*U*^11^	*U*^22^	*U*^33^	*U*^12^	*U*^13^	*U*^23^
Lu	0.01296 (8)	0.01284 (9)	0.00985 (8)	0.00229 (5)	−0.00408 (6)	−0.00270 (5)
C1	0.0173 (17)	0.0136 (18)	0.0151 (17)	−0.0027 (14)	−0.0047 (14)	−0.0044 (14)
C2	0.0226 (18)	0.0141 (18)	0.0098 (16)	0.0007 (15)	−0.0071 (14)	−0.0021 (13)
C3	0.028 (2)	0.045 (3)	0.019 (2)	0.019 (2)	−0.0110 (17)	−0.0139 (19)
C4	0.029 (2)	0.044 (3)	0.018 (2)	0.019 (2)	−0.0156 (17)	−0.0114 (18)
C5	0.0167 (17)	0.0183 (19)	0.0120 (17)	0.0024 (15)	−0.0052 (14)	−0.0030 (14)
C6	0.0208 (18)	0.0151 (18)	0.0139 (17)	−0.0003 (15)	−0.0035 (14)	−0.0031 (14)
C7	0.025 (2)	0.026 (2)	0.0193 (19)	−0.0135 (17)	0.0027 (16)	−0.0073 (17)
C8	0.030 (2)	0.026 (2)	0.0144 (18)	−0.0078 (17)	0.0006 (16)	−0.0094 (16)
C9	0.0171 (17)	0.0183 (19)	0.0174 (18)	0.0041 (15)	−0.0090 (15)	−0.0044 (15)
O1	0.0204 (13)	0.0277 (15)	0.0116 (12)	−0.0002 (11)	−0.0095 (10)	−0.0013 (11)
O2	0.0206 (13)	0.0245 (15)	0.0173 (13)	0.0044 (11)	−0.0081 (11)	−0.0104 (11)
O3	0.0177 (13)	0.0170 (13)	0.0136 (12)	0.0010 (11)	−0.0007 (10)	−0.0007 (10)
O4	0.0283 (14)	0.0213 (14)	0.0175 (13)	0.0011 (12)	−0.0058 (11)	−0.0106 (11)
O5	0.0182 (13)	0.0148 (14)	0.0283 (15)	0.0031 (11)	−0.0061 (11)	−0.0001 (11)
O6	0.0191 (13)	0.0207 (14)	0.0191 (14)	0.0063 (11)	−0.0027 (11)	0.0018 (11)
O7	0.0155 (12)	0.0155 (14)	0.0297 (15)	0.0023 (11)	−0.0059 (11)	−0.0004 (11)

### Geometric parameters (Å, °)

**Table:**

Lu—O1	2.825 (3)	C4—H4	0.9300
Lu—O1^i^	2.304 (3)	C5—O4	1.249 (4)
Lu—O2	2.297 (2)	C5—O3	1.269 (4)
Lu—O3	2.259 (2)	C5—C6	1.501 (5)
Lu—O4^ii^	2.195 (2)	C6—C7	1.384 (5)
Lu—O5	2.303 (3)	C6—C8	1.385 (5)
Lu—O6^iii^	2.313 (3)	C7—C8^v^	1.386 (5)
Lu—O7	2.272 (3)	C7—H7	0.9300
C1—O1	1.254 (5)	C8—H8	0.9300
C1—O2	1.273 (4)	C9—O6	1.250 (4)
C1—C2	1.501 (5)	C9—O5	1.253 (5)
C2—C4	1.382 (5)	C9—C9^iii^	1.540 (7)
C2—C3	1.383 (5)	O7—H7B	0.8484
C3—C4^iv^	1.383 (5)	O7—H7A	0.8520
C3—H3	0.9300		
			
O4^ii^—Lu—O3	99.39 (10)	C3—C2—C1	120.6 (3)
O4^ii^—Lu—O7	102.84 (10)	C2—C3—C4^iv^	120.9 (4)
O3—Lu—O7	141.27 (9)	C2—C3—H3	119.5
O4^ii^—Lu—O2	159.72 (10)	C4^iv^—C3—H3	119.5
O3—Lu—O2	84.59 (9)	C2—C4—C3^iv^	120.4 (4)
O7—Lu—O2	85.32 (10)	C2—C4—H4	119.8
O4^ii^—Lu—O5	79.68 (10)	C3^iv^—C4—H4	119.8
O3—Lu—O5	145.37 (10)	O4—C5—O3	124.3 (3)
O7—Lu—O5	70.51 (9)	O4—C5—C6	118.3 (3)
O2—Lu—O5	85.80 (9)	O3—C5—C6	117.4 (3)
O4^ii^—Lu—O1^i^	82.38 (10)	C7—C6—C8	120.0 (3)
O3—Lu—O1^i^	80.24 (9)	C7—C6—C5	120.1 (3)
O7—Lu—O1^i^	71.85 (9)	C8—C6—C5	119.9 (3)
O2—Lu—O1^i^	117.89 (9)	C6—C7—C8^v^	120.8 (4)
O5—Lu—O1^i^	133.05 (9)	C6—C7—H7	119.6
O4^ii^—Lu—O6^iii^	79.30 (10)	C8^v^—C7—H7	119.6
O3—Lu—O6^iii^	75.54 (9)	C6—C8—C7^v^	119.2 (4)
O7—Lu—O6^iii^	139.58 (9)	C6—C8—H8	120.4
O2—Lu—O6^iii^	82.52 (10)	C7^v^—C8—H8	120.4
O5—Lu—O6^iii^	70.26 (9)	O6—C9—O5	127.0 (3)
O1^i^—Lu—O6^iii^	146.60 (10)	O6—C9—C9^iii^	116.9 (4)
O4^ii^—Lu—O1	150.40 (9)	O5—C9—C9^iii^	116.1 (4)
O3—Lu—O1	70.43 (8)	C1—O1—Lu^i^	167.2 (2)
O7—Lu—O1	74.62 (8)	C1—O1—Lu	81.1 (2)
O2—Lu—O1	49.52 (8)	Lu^i^—O1—Lu	111.31 (9)
O5—Lu—O1	124.75 (8)	C1—O2—Lu	105.5 (2)
O1^i^—Lu—O1	68.69 (9)	C5—O3—Lu	139.5 (2)
O6^iii^—Lu—O1	122.18 (8)	C5—O4—Lu^ii^	157.9 (3)
O1—C1—O2	120.9 (3)	C9—O5—Lu	117.4 (2)
O1—C1—C2	121.4 (3)	C9—O6—Lu^iii^	116.6 (2)
O2—C1—C2	117.6 (3)	Lu—O7—H7B	125.1
C4—C2—C3	118.7 (3)	Lu—O7—H7A	119.5
C4—C2—C1	120.6 (3)	H7B—O7—H7A	105.1
			
O1—C1—C2—C4	−9.2 (6)	O6^iii^—Lu—O1—Lu^i^	144.41 (10)
O2—C1—C2—C4	166.0 (4)	O1—C1—O2—Lu	20.1 (4)
O1—C1—C2—C3	175.1 (4)	C2—C1—O2—Lu	−155.1 (2)
O2—C1—C2—C3	−9.7 (5)	O4^ii^—Lu—O2—C1	161.4 (3)
C4—C2—C3—C4^iv^	0.6 (7)	O3—Lu—O2—C1	58.9 (2)
C1—C2—C3—C4^iv^	176.5 (4)	O7—Lu—O2—C1	−83.7 (2)
C3—C2—C4—C3^iv^	−0.6 (7)	O5—Lu—O2—C1	−154.4 (2)
C1—C2—C4—C3^iv^	−176.5 (4)	O1^i^—Lu—O2—C1	−17.1 (3)
O4—C5—C6—C7	−151.7 (4)	O6^iii^—Lu—O2—C1	135.0 (2)
O3—C5—C6—C7	29.5 (5)	O1—Lu—O2—C1	−9.9 (2)
O4—C5—C6—C8	28.4 (6)	O4—C5—O3—Lu	31.2 (6)
O3—C5—C6—C8	−150.4 (4)	C6—C5—O3—Lu	−150.1 (3)
C8—C6—C7—C8^v^	0.5 (7)	O4^ii^—Lu—O3—C5	−46.2 (4)
C5—C6—C7—C8^v^	−179.4 (4)	O7—Lu—O3—C5	−170.7 (3)
C7—C6—C8—C7^v^	−0.5 (7)	O2—Lu—O3—C5	113.7 (4)
C5—C6—C8—C7^v^	179.5 (4)	O5—Lu—O3—C5	39.2 (4)
O2—C1—O1—Lu^i^	177.2 (9)	O1^i^—Lu—O3—C5	−126.7 (4)
C2—C1—O1—Lu^i^	−7.8 (13)	O6^iii^—Lu—O3—C5	30.1 (4)
O2—C1—O1—Lu	−15.8 (3)	O1—Lu—O3—C5	162.6 (4)
C2—C1—O1—Lu	159.2 (3)	O3—C5—O4—Lu^ii^	49.5 (9)
O4^ii^—Lu—O1—C1	−164.1 (2)	C6—C5—O4—Lu^ii^	−129.2 (6)
O3—Lu—O1—C1	−90.1 (2)	O6—C9—O5—Lu	−167.0 (3)
O7—Lu—O1—C1	106.9 (2)	C9^iii^—C9—O5—Lu	12.5 (5)
O2—Lu—O1—C1	9.8 (2)	O4^ii^—Lu—O5—C9	68.3 (3)
O5—Lu—O1—C1	54.6 (2)	O3—Lu—O5—C9	−23.3 (3)
O1^i^—Lu—O1—C1	−176.9 (3)	O7—Lu—O5—C9	176.0 (3)
O6^iii^—Lu—O1—C1	−32.5 (2)	O2—Lu—O5—C9	−97.5 (3)
O4^ii^—Lu—O1—Lu^i^	12.9 (2)	O1^i^—Lu—O5—C9	137.5 (2)
O3—Lu—O1—Lu^i^	86.88 (11)	O6^iii^—Lu—O5—C9	−13.9 (3)
O7—Lu—O1—Lu^i^	−76.14 (11)	O1—Lu—O5—C9	−130.0 (2)
O2—Lu—O1—Lu^i^	−173.24 (16)	O5—C9—O6—Lu^iii^	−167.5 (3)
O5—Lu—O1—Lu^i^	−128.40 (11)	C9^iii^—C9—O6—Lu^iii^	13.0 (5)
O1^i^—Lu—O1—Lu^i^	0.0		

Symmetry codes: (i) −*x*+1, −*y*, −*z*+2; (ii) −*x*+1, −*y*+1, −*z*+2; (iii) −*x*, −*y*+1, −*z*+2; (iv) −*x*+1, −*y*, −*z*+1; (v) −*x*+2, −*y*+1, −*z*+1.

### Hydrogen-bond geometry (Å, °)

**Table:**

*D*—H···*A*	*D*—H	H···*A*	*D*···*A*	*D*—H···*A*
O7—H7A···O3^i^	0.85	1.92	2.752 (5)	167
O7—H7B···O2^vi^	0.85	1.92	2.764 (6)	177

Symmetry codes: (i) −*x*+1, −*y*, −*z*+2; (vi) −*x*, −*y*, −*z*+2.
